# Some Common Skin Affections of the Face

**Published:** 1907-06-01

**Authors:** G. Norman Meachen

**Affiliations:** Physician for Skin Diseases, the Prince of Wales's General Hospital, etc.


### Hospital Clinics.

### SOME COMMON SKIN AFFECTIONS OF THE FACE.

By G. NORMAN MEACHEN, M.D., B.S., M.R.C.P., Physician for Skin Diseases, the Prince of Wales's General Hospital, etc.

A Lecture delivered at the North-East London Post-Graduate College.

Gentlemen,—Such is human nature that many a skin eruption will be cheerfully borne, or, at any rate, tolerated, as long as the face is spared. Once let the complexion be affected, especially in sensitive individuals, and the demand for speedy relief is at once loud and persistent. The same feeling which leads the wounded animal to avoid the company of its own species and to hide away in solitary retirement, and also induces man, when his countenance is no longer pleasant to look upon, to shrink from society and to cover over any facial defect or blemish as efficiently as possible.

There are several reasons why the face is specially liable to cutaneous disorders. It is continually being exposed to the atmosphere with its contained impurities and to varying climatic conditions—to the scorching rays of the sun or to the biting east wind. For cosmetic purposes it is subjected by many people to extraordinary and wholly unnatural treatment with soaps, washes, and powders, many of which may be highly irritating. The process of shaving, in the male, opens the door to endless possibilities of infection. Moreover, from the anatomical standpoint, the skin of the face presents some peculiarities. The epidermis is relatively thin, while the sub-epidermic plexus of blood-vessels contains a great number of capillaries. To the richness of the vascular supply of this region is owing the rapidity with which wounds of the surface heal, while, at the same time, the intimate connection that exists between the cutaneous vessels and those of the stomach is responsible for many of the symptoms—*e.g.*, flushing after meals, which characterise diseases of the skin affecting this part of the body.

### A Classification of Facial Skin Lesions.

For practical purposes, we may classify the facial dermatoses under four main headings:—(1) Congestive; (2) inflammatory; (3) parasitic; and (4) neoplastic.

In the first group we must place those affections which arise from the absorption of some toxic material from the alimentary canal, or which proceed from a disordered state of the stomach. These are certain of the erythemata and also *rosacea* in its early stages. Morbid flushing after food or taking hot liquids is frequently associated with, and furnishes an indication of, a condition of chronic gastritis or flatulent dyspepsia. This may ultimately develop into a more or less pronounced rosacea which later may exhibit hypertrophic changes, together with the formation of teleangiectases. From such a state it is but a small step to actual inflammatory change. We then get definite acneiform papules or pustules super-added, and in the more advanced cases the involved skin becomes chronically indurated. I do not mean to say that all cases of rosacea are dependent upon gastric disorders, but it is very common to obtain in such a history of indigestion and especially of constipation. The connection of this affection with chronic indulgence in alcoholic liquors is popularly supposed to be very intimate, but I have already shown that many of the sufferers therefrom are quite innocent of this form of intemperance.* Constitutional treatment is absolutely essential in all cases, and without it, local applications will signally fail. Calamine lotion is useful in cases where there is much inflammation, otherwise weak solutions of ichthyol may be applied, after which ointments containing 20 grains of ichthyol or precipitated sulphur, preferably the former, with 10 grains of zinc oxide in an ounce of lanoline or vaseline, are lightly smeared over the affected parts. For immediate results, painting with a solution of adrenalin, as suggested by W. J. Munro, may give a temporary anæmia. Any pustules require treating antiseptically.

### Common Acne.

Common *acne* is, perhaps, one of the most frequent, and when severe, is also a most disfiguring affection of the facial integument. The familiar "blackhead" is a source of much annoyance to adolescents, and many are the despairing attempts to extract them by the traditional watchkey, but it more often than not happens that, by unskilful manipulation, suppuration is induced in the follicle, leading to the development of an ugly pustule. The sudden outbreaks of acne upon the forehead after eating a quantity of biscuits or rich puddings are rather curious, and make one think that the pathological basis of the affection is, perhaps, something more than a mere blockage of the pilo-sebaceous follicle. A large number of the cases are closely connected with seborrhœa of the scalp, and the researches of Gilchrist, of the Johns Hopkins University, who found in the *Bacillus acnes* in the lesions, and this micro-organism, by the way, has been shown to be identical with the micro-bacillus of seborrhœa (Sabouraud), a fact which serves to explain this close relationship. The acne-pustule, of course, contains pyogenic cocci. Acne vulgaris is not necessarily dependent upon any gastric derangement, though constipation is a common enough accompaniment of the disorder. Local treatment is nearly always successful, especially before the pustular stage is reached. All the comedones should be extracted with a proper extractor, of which many good patterns are sold. The face may be well steamed at night, and may afterwards be gently rubbed with a flannel and good soft soap. A lotion of

* "Brit. Journ. of Inebriety," April 1904, p. 274.

sulphur or ichthyol may then be applied. It is a good practice to touch any obstinate papule with a pointed wooden match dipped in pure phenol, or, if preferred, in a mixture of equal parts of powdered camphor and phenol which does not leave a white mark, and also to open any pustule that may be present. Both the *x*-ray and the vaccine treatment of acne should, I think, be reserved for those cases where all the ordinary remedies have been well tried and have not proved successful.

### Eczema of the Face.

*Eczema* attacks the face in many different ways. In infants we have the familiar impetiginous variety with much weeping and crust-formation, the latter produced largely from rubbing and chafing. Then we see the small, disc-like, nummular forms of eczema squamosum, so common in school children. This type is frequently mistaken for ringworm, but a surface-scraping shows no spores or mycelium. In adults the acute erythematous variety is, perhaps, more frequent than papular forms. At all ages the condition may be accompanied by seborrhœa capitis, and it is rather a useful thing to know that the post-auricular region is nearly always involved if the case be primarily one of seborrhœa. Nasal catarrh or rhinitis furnishes another form of eczema, which is, at first, localised around the anterior nares, but which soon spreads to adjacent parts of the face. It is sometimes most difficult to determine which is the primary condition in the case of a very eczematous-looking ear—an otorrhœa or an auricular eczema. Again, some cases of so-called erysipelas of the face are really nothing more than an acute erythematous eczema. A curious sort of eczema, half-dermatitis and half seborrhœa, is often seen upon the forehead and temples, encroaching upon the hairy scalp, and resulting from irritating hat-bands. Whatever be the type of the disease, we have first to consider what is the underlying cause, and then to soothe any inflammatory process that may be present. The seborrhœic cases require the special treatment of the scalp which I have previously discussed here. The best all-round lotion is one of calamine, which should not contain much glycerine if the eczema be acute and weeping. The lotio plumbi may be added to it with advantage, and as the case progresses towards recovery, a little weak cyllin or a few drops of the liquor carbonis detergens may be used instead. The oleates of bismuth and zinc with the oxide of the latter metal, and the acetate of lead, alone are permissible in the acuter stages, but a little ichthyol may be allowed later. If the impetiginous element predominates, five to ten grains of the ammoniated mercury may be added to the ointment. No soap must be employed at all, but bran or oatmeal may be used in warm water with a little fresh cow's milk for bathing the affected parts.

### Parasitic Affections.

Many of the strictly parasitic affections of this region may, of course, be much inflamed, as, for example, *sycosis* due to the tricophyton fungus. As a rule, however, hyphogenic sycosis is characterised by more lumpiness and less pustulation than the coccigenic variety. The affected hairs in the beard or moustache region will generally come out easily upon gentle traction, and their sheaths may appear thickened even to the naked eye, whilst under the microscope the fungus can be identified and stained if necessary. It is necessary to exercise some caution in the diagnosis of sycosis, as syphilis may present a very similar appearance; hence, it is well to inspect other regions of the body, such as the tongue, the shins, etc., and to inquire if there are any other patches elsewhere. On the other hand, a non-parasitic sycosis may be accompanied by much seborrhœa of the scalp or adjacent hairy parts of the face. For ringworm of the beard, the dilute nitrate of mercury ointment is useful, combined with clipping short of the affected hairs and their epilation, as far as possible. In coccigenic sycosis, antiseptic lotions of cyllin and ointments of sulphur, carbolic and salicylic acids are good applications.

The common circular patches of *tinea circinata* upon the forehead or chin are easily cured by rubbing in ammoniated mercury ointment, before which one application of tincture of iodine may be made. At the same time it is important to examine the scalp very carefully for unsuspected patches of tinea tonsurans. Among the neoplastic affections *lupus vulgaris* is one of the most disfiguring. It requires to be carefully distinguished from *syphilis*, as both these affections present very similar appearances; they each cause loss of tissue, and leave scars. Lupus vulgaris may occur at any age and does not tend to such great destruction of tissue as does syphilis. On pressing out the blood from the part with a piece of plain glass (a watch-glass or a lens), there can always be seen in lupus the characteristic yellowish-brown areas or nodules, which have been likened so aptly to "apple-jelly." In syphilis these are not present, and there will generally be other evidences of the disease elsewhere, such as old scarring, quite apart from any history, which may be absolutely worthless. In the child we frequently see lupus attacking those of the nervous disposition, with blue sclerotics, fine silky hair, and general frailty, though there are, of course, exceptions. The nose is most liable to be attacked, and then, perhaps, the side of the face in front of the ear, the skin around the mouth and below the eye, in order of frequency. Ordinary lupus may become inflamed, and may then weep just like an acute eczema, or it may be much crusted over, in which case it may resemble a severe impetigo. The length of time that the condition has been present should alone suffice to prevent any confusion with the latter comparatively simple complaint, even apart from a close examination.

The treatment of the tuberculous affections in this region is a big subject. There are those who still maintain that excellent results may be obtained from the older methods, such as scraping, scarification, or cauterising, combined with skin-grafting, if necessary, and I can only say that I have seen and undertaken such operations with success. Recurrence may take place, however, even with the newest methods, but against this fact we have the much better cosmetic results obtained by means of the Finsen light and the *x*-rays. Each case must be taken on its own merits, and whereas total excision of the affected area might be practicable in one case, in another, exposure to the *x*-rays under the hands of a competent medical radiographer may be the very best form of treatment.

### Ulcerations.

I will mention *lupus erythematosus* here on account of the diagnosis between it and lupus vulgaris. It is unfortunate that the first name of both these affections is identical, for, while bearing in mind the relationships of lupus erythematosus to tuberculosis, this disease has nothing like the terrors of its malignant cousin; in fact, there are many points in its pathology which tend to separate it from true lupus and to cause it to be grouped among the toxic erythemata. It is not often seen before the age of 25, and it usually begins upon the centre of the nose, spreading laterally in one or both directions in the shape of the well-known "bat's wing." The centre is slightly atrophic, but never so markedly cicatricial as in lupus vulgaris, while the advancing border is reddened, slightly scaly, and elevated above the surrounding skin. A better name for this disease is that of Unna's, namely, "Ulerythema centrifugum." The treatment consists in the application of a mild caustic to the advancing border—the mixture of camphor and phenol referred to previously is an excellent paint for this purpose—and in the administration of quinine internally. This drug may be given in full doses, or, if it should not be well borne, then salicin may be tried instead. The bowels should be kept freely open.

*Rodent ulcer* is another affection of malignant character—I use the term "malignant" here in its specific sense—which commonly attacks the face, the favourite situation being the parts about the internal canthus, and the naso-labial junction. When fully developed the condition should not be easily missed, the characteristically rolled edge, the long duration, and the situation, being the chief points in the diagnosis. A microscopic examination of a portion of the edge will make things absolutely certain. In this affection I think we have in the *x*-rays a really reliable remedy, though a more recent method of cure consists in the electrolytic application of zinc ions. A full description of this method, by Dr. Lewis Jones, was given in The Hospital of October 20, 1906.

